# Evidence on the Porter hypothesis: China’s Resource Tax Law may be a path to achieve corporate sustainable development

**DOI:** 10.1371/journal.pone.0323668

**Published:** 2025-05-30

**Authors:** Xiaoqi Zhang, Yu He

**Affiliations:** School of Finance, Chongqing Technology and Business University, Nan’an District, Chongqing, China; Universiti Brunei Darussalam, BRUNEI DARUSSALAM

## Abstract

Promoting utilization efficiency and sustainable energy development is crucial for achieving sustainable social development in China, the largest energy producer and consumer worldwide. Accordingly, the Chinese authorities enacted the Resource Tax Law (RTL) on August 26, 2019. Whether such a policy can achieve its desired goals has not been verified. Thus, our study applies a difference-in-differences approach to examine the effect of RTL on a firm’s energy efficiency (EE) with panel data of A-shares from 2017 to 2022. Benchmark analysis and robustness tests demonstrate RTL’s significant effect on resource-based firm’s EE. Additional tests deeply explore the policy effect under different situations. First, RTL boosts more for firms with higher initial EE levels than those with lower levels. Second, RTL enhances EE by promoting firms’ research and development investments, confirming the existence of the Porter hypothesis in China. Third, this policy shows a stronger positive effect on firms with political connections than those without. Finally, we detect that RTL has no significant impact in the western region, while its effect is significantly stronger in the middle region compared to the eastern region. Our empirical findings suggest that corporate firms and the government should adopt appropriate actions to enhance RTL’s policy effect.

## 1. Introduction

The resource-intensive development model has brought China high economic growth but has also led to increasingly serious environmental problems. China’s total energy consumption was 4.64 billion tons of standard coal in 2018, which accounted for 23.6% of the global total consumption and ranked first for the tenth consecutive year worldwide (China Energy Supply and Demand Report). Moreover, China’s energy consumption per unit of gross domestic product was 347.6 TCE/MD (tons of standard coal equivalent per million US dollars), 1.5 times the global average. Regarding advanced economies, the United States (US) was at 160.3 TCE/MD, Japan was at 128 TCE/MD, the United Kingdom was at 97.9 TCE/MD, and Germany was at 105.7 TCE/MD (Integrated Energy Services Plaza, IESPLAZA). Therefore, China’s energy efficiency (EE), reflecting economic output per unit of energy input, is relatively low, with issues of resource wastage and low utilization rates. Chinese authorities enacted the Resource Tax Law (RTL) on August 26, 2019, to improve resource utilization efficiency, reduce energy consumption per unit, and shift the country’s development mode from extensive to sustainable economic development.

RTL enforcement represents a significant government effort to change the economic development model. Considering the policy’s profound implications, scholars have attempted to investigate its effects on the economy and environment [1–10]; however, the conclusions remain controversial. As RTL directly affects firms, whether this policy can promote firms’ technological progress and further improve their EE is a meaningful but unexplored question in the current literature. RTL has been implemented for over 3 years, its effect is fully reflected, and related data are sufficient for empirical research. Therefore, we aim to fill the above literature gap and explore the effect of RTL on a firm’s EE.

According to the RTL, organizations and individuals developing taxable resources in the territories and maritime areas under Chinese jurisdiction are taxpayers. Moreover, downstream firms of taxpayers are also affected due to the shifted costs. Thus, we categorize our sample into resource-based firms(treatment group) and non-resource-based firms (control group). Resource-based firms refer to the taxpayers of RTL and those heavily relying on taxable resources for producing (i.e., downstream firms of taxpayers). Those firms are mainly affected by RTL. We first conduct a parallel trend test to check whether a common (discrete) trend exists prepolicy (postpolicy). Then, a difference-in-differences (DID) approach is utilized as our benchmark analysis to investigate the effect of RTL on A-share listed firms’ EE from 2017 to 2022. Several tests, including PSM-DID, counterfactual and in-space placebo tests, are adopted to verify the main result. Finally, we conduct additional tests, including quantile analysis, mediating effect analysis and heterogeneous impacts, to investigate the policy effect under different situations.

We achieve several findings with the above empirical analyses, as shown below. a) Our benchmark analysis demonstrates that RTL significantly enhances resource-based firms’ EE, which holds after several robustness tests. b) Quantile analysis shows RTL boosts more for firms with higher initial EE levels than others with lower levels. c) Mediating effect analysis shows that RTL promotes firms’ EE through research and development (R&D) investments. d) The heterogeneous impacts show a stronger positive effect of RTL in the PC firms and in the middle region.

The main contributions are summarized as follows. a) Our study examines the policy effect of RTL from a micro perspective (i.e., firms’ EE), which provides new evidence on the positive effect of RTL on resource-based firms and thus fills the research gap. b) Firms with lower initial EE levels, which should mostly be improved, are less positively affected; thus, the authorities should offer these firms preferential policies to improve their EE. c) Our study demonstrates the existence of the innovation compensation effect created by RTL, confirming the existence of the Porter hypothesis in China. d) We also prove the necessity for firms to establish PC with the government, as these firms can better benefit from enforcing RTL. e) Our empirical results suggest that policymakers should offer differentiated policies for firms in different areas to improve RTL’s policy effect and promote sustainable development nationwide.

## 2. Literature review and hypothesis development

### 2.1. Literature review

The Porter hypothesis proposes that an effective environmental policy can stimulate innovation, enhance firm competitiveness, and thereby promote sustainable development [11]. In recent years, many countries have adopted environmental policies to promote sustainable social development. Scholars found that the effects of those policies on EE are not always as positive as policymakers expected, and the relevant conclusions remained controversial.

Several scholars support the Porter hypothesis, observing positive effects of environmental policies on EE, see, for example, Segarra-Blasco and Jové-Llopis (2019), Xu and Xu (2022), Zhang et al. (2020), Li et al. (2023), Zhao et al. (2020), Sun et al. (2021), Paramati et al. (2022), Wang et al. (2023), Wang et al. (2020), Pan et al. (2019), Yang et al. (2023b), Song et al. (2023), He and Wang (2024), and Zhang and Wang (2024). Pan et al. (2019) pointed out that environmental regulations, including both market-based (MER) and command and control (CCER), could promote EE [12]. After surveying 8,213 firms in European countries, Segarra-Blasco and Jové-Llopis (2019) found that regulations and cost savings are more effective in promoting EE [13]. Wang et al. (2020) stated that policy effects are the main factors driving renewable energy consumption growth in the G20 high-income countries. Moreover, as incomes rose, policy and environmental pressures became increasingly important in promoting renewable energy [14]. Zhang et al. (2020) showed that environmental regulation could promote capital substitution for energy and achieve an energy-saving effect in the long run [15]. Zhao et al. (2020) demonstrated that energy-saving policies positively impact EE, suggesting that economic incentive is the most significant factor in improving EE [16]. Technological innovation positively impacted neighboring regions’ EE performance, and environmental technology played a key role in reducing energy consumption and promoting EE [17,18]. Xu and Xu (2022) proposed that incentive environmental regulations substantially affect regions with higher EE, while mandatory regulations are more suitable for others with lower EE [19]. Yang et al. (2023b) and Wang et al. (2023) revealed that the marketization of urban land transfer and the low-carbon city pilot policy could improve EE through technological innovation and industrial structure optimization [20,21]. The digital economy, the establishment of environmental courts, the green credit policy, and the carbon emissions trading system were all found to have significant positive effects on EE [6,22–24].

Conversely, other scholars argued that environmental policy’s effect on a firm’s EE is uncertain, as such policies may increase environmental costs, potentially harming overall efficiency (García-Quevedo and Jové-Llopis, 2021; Guo and Yuan, 2020; Wang et al., 2022b; Wu et al., 2020; Ngo, 2022; Curtis and Lee, 2019; Hao et al., 2022; Zhu et al., 2024). Specifically, Curtis and Lee (2019) explored how factories adjust onsite power generation when constrained by environmental regulations. The results showed that the EE of onsite power generation could improve (reduce) for the producers limited by quota trading (restricted by nitrogen oxide directive regulations) [25]. Zhu et al. (2024) found that environmental regulation and EE remain a“U”shaped relationship, but industrial structure optimization is conducive to weakening the initial inhibitory effect of environmental regulation [26]. Consider the Chinese government’s regulation of EE, Yang et al. (2020) discovered an inverse U-shaped relationship between market segmentation and EE [27]. Guo and Yuan (2020) and Wu et al. (2020) detected a nonlinear relationship between environmental policies and green total factor EE. Moreover, MER in China was more effective than CCER, which exceeded the optimal level [28,29]. García-Quevedo and Jové-Llopis (2021) found that environmental taxes, tax exemptions, and regulations could not significantly affect energy-saving investments, while subsidies promote energy-saving investments [30]. Ngo (2022) stated that when the values of MER and CCER exceed their respective levels, their impacts on the total factor EE index gradually increase (i.e., a U-shaped relationship) [31]. Wang et al. (2022b) and Hao et al. (2022) showed that environmental regulations with different intensities might lead to various impacts of energy endowment and information and communication technology development on EE and GTFEE. CCER (MER) has an insignificant (a positive) moderating effect on the relationship between energy endowment and EE [32,33].

Although relevant studies examined the impact of environmental policies on EE, the Porter Hypothesis remains debated. Moreover, In the pursuit of sustainable development, the effect of policies, especially RTL, on resource-based firms’ EE has been not examined. Therefore, studying whether the RTL stimulates innovation and improves EE in resource-based firms provides valuable new evidence for the Porter Hypothesis.

### 2.2. Policy review and hypothesis development

In 1984, the Chinese authorities first promulgated the Resource Tax Regulation (Draft) to levy resource tax. After that, this administrative regulation was adjusted several times; however, its role in protecting resources and promoting rational resource development has not yet been fully realized. Thus, the central government enacted RTL on August 26, 2019, to improve resource utilization efficiency and promote sustainable social development. Compared to the previous administrative regulation, RTL has a) standardized the relationship between taxes and fees, b) standardized tax rates and tax items, c) standardized the administration of tax reductions and exemptions, d) established the taxation of AD valorem (main) and volume-based (auxiliary), and e) granted local governments greater tax administration powers.

Given the discussion in Section 2.1, we realize the significant effect of environmental policies on firms’ EE, although the direction of this effect remains controversial. Moreover, according to RTL, organizations and individuals that exploit taxable resources in the territories and maritime areas under Chinese jurisdiction are taxpayers. Downstream firms of taxpayers are also affected due to the shifted costs. We thus believe that RTL may affect resource-based firms’ EE. In addition, RTL encourages firms to green innovation, production process improvement, and enhance resource utilization efficiency [4]. Technological innovation can significantly reduce energy consumption and improve EE [16,17]. Thus, RTL may create an innovation compensation effect by promoting corporate R&D investment, which affects the firm’s EE. To test these conjectures, we develop the following hypotheses:

**H1:** RTL significantly affects on resource-based firms’ EE.

**H2:** RTL affects resource-based firms’ EE through R&D investments.

## 3. Data and methodology

### 3.1. Data and sample

This study has two primary data sources: a) firm energy consumption data from the Environmental Survey and Reporting database and b) other data from the China Stock Market and Accounting Research database. This study uses 2017–2022 as the sample period because a) most of the data are only available until 2022, and b) RTL was enacted on August 26, 2019. Therefore, we chose 3 years preevent and postevent. All Chinese A-shares are included in our initial sample. For the reliability of the data analysis, we screened our sample firms according to the criteria listed in Table 1. Our final sample contains 2,590 firms.

**Table 1:** Data filtration process.

Criterion	Reason
a) Excluding financial firms	These firms are subject to different regulations.
b) Excluding firms listed on the Growth Enterprises Market and the Science and Technology Innovation Board Market	Control for the potential effect of life cycle.
c) Excluding firms in special treatment (ST, *ST, PT)	Our study focuses on general rather than special cases.
d) Excluding each firm’s first-year observations	Control for the IPO effect.
e) Excluding observations with missing values	We need necessary data for empirical analyses.
f) All continuous variables are winsorized at 1%	Control the potential effect of extreme values.

This table presents the data filtration process.

### 3.2. Variables

#### 3.2.1. Dependent variable.

As discussed, EE is the dependent variable of this study. Based on Liu et al. (2023), Song et al. (2022b), Yang et al. (2023a), and Du et al. (2022) [34–37], we use Eq 1 to calculate the firm’s EE.


$${EE}_{i,t}=\;\frac{{Output\;(Total\;Operating\;Income)}_{i,t}}{{Energy\;Consumption\;(Total\;Standard\;Coal\;Consumption)}_{i,t}}$$ (1)

Here, *Output* represents the total operating income. *Energy Consumption* is the total standard coal consumption, the sum of water consumption, electricity consumption, raw coal use, natural gas use, gasoline use, diesel use, and centralized heating multiplied by the corresponding conversion coefficients. Furthermore, *i* and *t* are firm i and year t, and *EE* stands for firm’s EE, which is Chinese yuan (CNY) per ton of standard coal. Thus, the larger the value, the better the EE and the stronger the firm’s sustainability.

#### 3.2.2. Independent variables.

The core independent variable is RB*Time, which equals one for a resource-based firm in postevent and zero for others. Since RTL was enacted on August 26, 2019, we define 2017–2019 (2020–2022) as preevent (postevent). Moreover, resource-based firms are mainly affected by RTL. Therefore, we divide the sample firms into resource-based and others (i.e., treatment and control groups). Following Song et al. (2022a) [38], the resource-based sector includes 12 industries, as shown in Table 2. In addition, according to the Hausmann test, we use a fixed effect model (We do not report the Hausmann test result for brevity); therefore, time and grouping dummy variables are not controlled separately to prevent multicollinearity issues.

**Table 2:** Resource-based industries list.

Code	Industry	Code	Industry
B06	Coal mining and washing industry	C26	Chemical raw materials and chemical products manufacturing industry
B07	Oil and gas mining industry	C30	Non-metallic mineral products industry
B08	Ferrous metal mining and dressing industry	C31	Ferrous metal smelting and rolling processing industry
B09	Non-ferrous metal mining and dressing industry	C32	Non-ferrous metal smelting and rolling processing industry
B10	Non-metallic mining and dressing industry	C33	Metal products industry
C25	Petroleum processing and coking industry	D44	Power and heat production and supply industry

The above industrial code adopts the Classification of Industries of National Economy in 2012.

Regarding variables, we follow previous studies [36,37,39,40] and adopt two types of factors: corporate governance (*State Owner*, *FST*, and *Duality*) and financial fundamentals (*Leverage*, *Current*, and *Cash Flow*). Please refer to S1 File Appendix A for variable definitions.

### 3.3. Summary statistics

Table 3 shows that our sample includes 14,198 observations, with 2,909 and 11,289 observations in the treatment and control groups, respectively. Panel A shows that EE’s mean, min, and max values are 5.382, 2.054, and 10.052, respectively. RB*Time’s mean value is 0.108, indicating that 10.8% of observations belong to the treatment group and postevent. Panel B presents the *t*-test difference of means of main variables between treatment and control groups, where the treatment group’s *EE*, *State Owner*, *FST*, and *Leverage* (*Duality*, *Current*, and *Cash Flow*) are significantly higher (lower) than the control group.

**Table 3:** Summary statistics.

Panel A: Full sample						
Variables	Obs	Mean	Median	Std. Dev.	Min	Max
EE	14,198	5.382	5.231	1.463	2.054	10.052
RB*Time	14,198	0.108	0.000	0.311	0.000	1.000
State Owner	14,198	0.045	0.000	0.142	0.000	0.740
FST	14,198	2.546	1.396	3.352	0.231	25.040
Duality	14,198	0.261	0.000	0.439	0.000	1.000
Leverage	14,198	0.442	0.437	0.192	0.061	0.912
Current	14,198	2.109	1.581	1.792	0.265	13.929
Cash Flow	14,198	0.146	0.119	0.107	0.007	0.587
Panel B: RB Firms vs. Other Firms						
Variables	RB Firms		Others		*t*–test of difference	
	Obs	Mean	Obs	Mean	Difference	*t*-stat.
EE	2,909	5.728	11,289	5.292	0.436***	14.436
State Owner	2,909	0.058	11,289	0.042	0.016***	5.509
FST	2,909	2.761	11,289	2.491	0.270***	3.882
Duality	2,909	0.205	11,289	0.276	−0.071***	−7.832
Leverage	2,909	0.449	11,289	0.440	0.009**	2.230
Current	2,909	1.856	11,289	2.175	−0.319***	−8.587
Cash Flow	2,909	0.116	11,289	0.154	−0.038***	−17.116

This table presents the results of summary statistics. ***, **, and * refer to the significance at the 1%, 5%, and 10% levels, respectively (hereinafter inclusive).

## 4. Empirical analysis

### 4.1. Parallel trend analysis

A common (discrete) trend between the treatment and control groups’ preevent (postevent) is the basic premise of analyzing policy effects with a DID model. Therefore, we adopt regression analysis to examine the applicability of this method by constructing the following Eq 2:


$${EE}_{i,t}=\alpha_{i,t}+\;\beta_{i,t}^{1}{RB2018}_{i,t}+\beta_{i,t}^{2}{RB2019}_{i,t}+\ldots +\beta_{i,t}^{7}{RB2022}_{i,t}+\theta_{i,t}{Controls}_{i,t}+\;\mu_{i\;}{{+v}_{t}+\varepsilon }_{i,t}$$ (2)

where ${EE}_{i,t}$ is the dependent variable. ${RB2018}_{i,t}$, ${RB2019}_{i,t}$, …, ${RB2022}_{i,t}$ are independent variables. ${RB2018}_{i,t}$ equals one for a resource-based firm in 2018, zero for others, and so on; *Governance* (*State Owner*, *FST*, and *Duality*), and *Fundamentals* (*Leverage*, *Current*, and *Cash Flow*) are the control variables. $\mu_{i}$*,* $\nu_{t}$ *and* $\varepsilon_{i,t}$ represent firm fixed effects, time fixed effects and random disturbance term.

**Fig 1:**
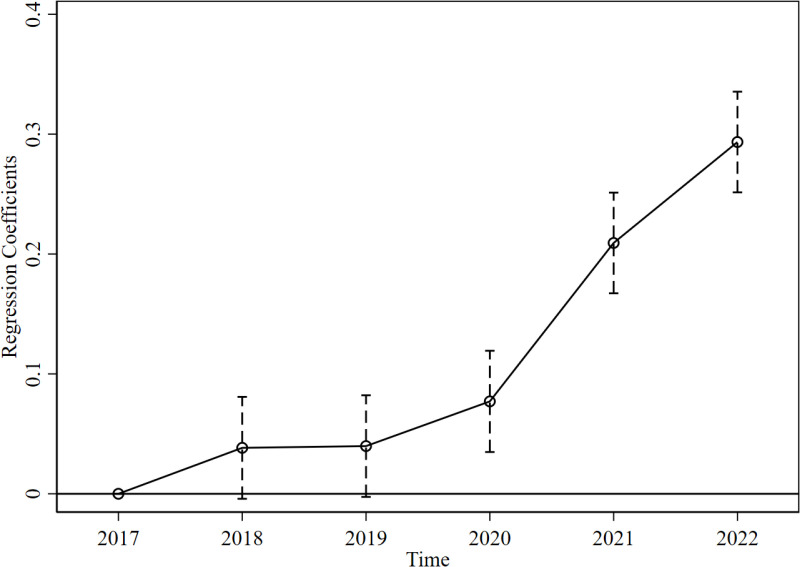
Parallel trend analysis. This figure shows the parallel trend. We categorize resource-based and other firms as treatment and control groups. Time 2017 serves as the baseline. The regression coefficients represent the policy effect, reflecting the differences between the treatment and control groups at each time points.

We draw the parallel trend (Fig 1) based on the result of Eq 2. The confidence intervals of the coefficients before (after) 2019 include (deviate from) zero, indicating a common trend (a significant difference) between the treatment and control groups before (after) 2019, thus supporting the applicability of the DID method. Furthermore, the coefficients show a rising trend, suggesting that the effect of RTL increases. Therefore, this policy should be considered for long-term implementation, which can better improve firms’ EE.

### 4.2. Benchmark analysis

We construct Eq 3 to test Hypothesis 1, i.e., exploring the impact of RTL on resource-based firms’ EE:


$${EE}_{i,t}=\alpha_{i,t}+\;\beta_{i,t}{RB*Time}_{i,t}+\;\theta_{i,t}{Controls}_{i,t}+\;\mu_{i}{{\;+\;v}_{t}\;+\;\varepsilon }_{i,t}$$ (3)

where RB*Time equals one for observations that are resource-based firms and post policy, and zero for others. Other variables are introduced in Eq 2.

This study conducts regression analysis by gradually adding control variables, and the results are consistent. Therefore, the issue of omitted variables, a type of endogeneity, is less likely to interfere with the accuracy of data analysis. Table 4 shows that RB*Time positively affects *EE*, meaning RTL significantly enhances resource-based firms’ EE. The above result confirms Hypothesis 1. Furthermore, *State Owner*, *Leverage*, and *Cash Flow* (*FST*, *Duality*, and *Current*) have significant positive (negative) effects on *EE*.

**Table 4:** Benchmark analysis.

	EE	EE	EE
RB*Time	0.159***	0.157***	0.166***
	(0.000)	(0.000)	(0.000)
Governance		Yes	Yes
Fundamentals			Yes
Fixed Effect	Yes	Yes	Yes
Observations	14,198	14,198	14,198
R-squared	0.040	0.046	0.085

This table presents the results of our benchmark analysis. The fixed effect model is adopted to control the time and firm fixed effect. Robust standard errors are used. ***, **, and * indicating significance at the 1%, 5%, and 10% levels, respectively. We add our proposed factors into the regression model step by step.

### 4.3. Robustness checks

**Fig 2:**
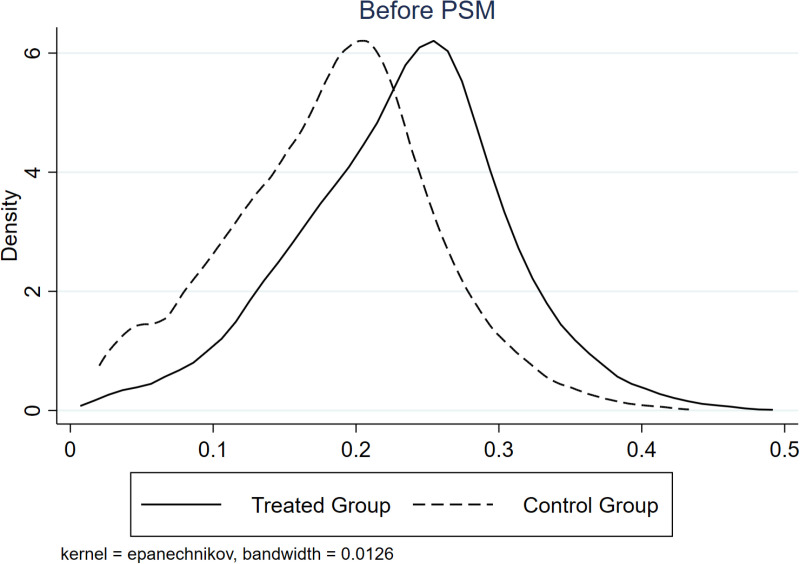
KDC before PSM. This figure shows the KDC before PSM. We categorize resource-based and other firms as treatment and control groups.

**Fig 3:**
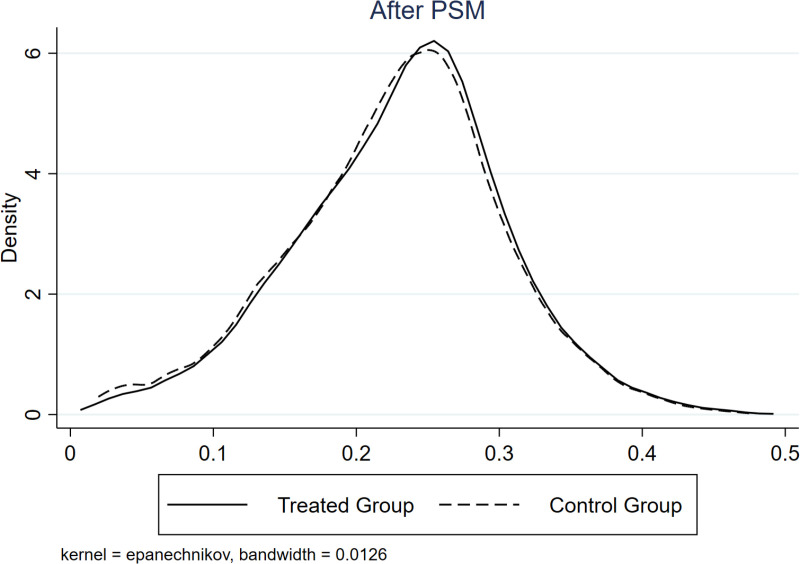
KDC after PSM. This figure shows the kernel density curve after PSM. We categorize resource-based and other firms as treatment and control groups.

We implement the following PSM-DID tests. First, we utilize the propensity score matching (PSM) DID approach and rerun regressions with Eq 3. Figs 2 and 3 shows the kernel density curves (KDC) of the treatment and control groups before (after) PSM, where the difference between these two groups decreases after PSM. Comparing Tables 5 and 4, RB*Time shows little changes in both coefficient size and significance level, indicating that the main finding of this study is robust.

**Table 5:** PSM-DID.

	EE	EE	EE
RB*Time	0.153***	0.154***	0.152***
	(0.000)	(0.000)	(0.000)
Governance		Yes	Yes
Fundamentals			Yes
Fixed Effect	Yes	Yes	Yes
Observations	5,814	5,814	5,814
R-squared	0.107	0.122	0.170

This table presents the results of placebo test - PSM-DID. For the PSM, the matching ratio is 1:1, the matching covariates are our control variables, and the matching method is nearest neighbor matching. The fixed effect model is adopted to control the time and firm fixed effect. Robust standard errors are used. ***, **, and * indicating significance at the 1%, 5%, and 10% levels, respectively. We add our proposed factors into the regression model step by step.

### 4.4. Placebo tests

#### 4.4.1. Counterfactual test.

To verify that the positive policy effect observed previously is due to the enforcement of RTL, we first adopt Eq 4 for a counterfactual test as follows:


$${EE}_{i,t}=\alpha_{i,t}+\beta_{i,t}{RB*Time1}_{i,t}+\theta_{i,t}{Controls}_{i,t}+\;\mu_{i}{{+v}_{t}+\varepsilon }_{i,t}$$ (4)

We replaced the sample period with 2017–2019 and assumed RTL was enforced in 2018. Therefore, *RBTime1* equals one for resource-based firms and postpolicy observations and zero for others; other variables are introduced in Eq 2. Table 6 shows that the coefficients *RBTime1* are insignificant, suggesting the main finding in the above section is robust.

**Table 6:** Counterfactual test.

	EE	EE	EE
RB*Time1	0.005	0.005	0.012
	(0.796)	(0.767)	(0.449)
Governance		Yes	Yes
Fundamentals			Yes
Fixed Effect	Yes	Yes	Yes
Observations	6,633	6,633	6,633
R-squared	0.029	0.034	0.129

This table presents the results of counterfactual test. The fixed effect model is adopted to control the time and firm fixed effect. Robust standard errors are used. ***, **, and * indicating significance at the 1%, 5%, and 10% levels, respectively. We add our proposed factors into the regression model step by step. The sample period of this test is from 2017 to 2019. RB*Time1 takes the value of one if the observation is a resource-based firm and after 2018, and zero otherwise.

#### 4.4.2. In-space Placebo Test.

**Fig 4:**
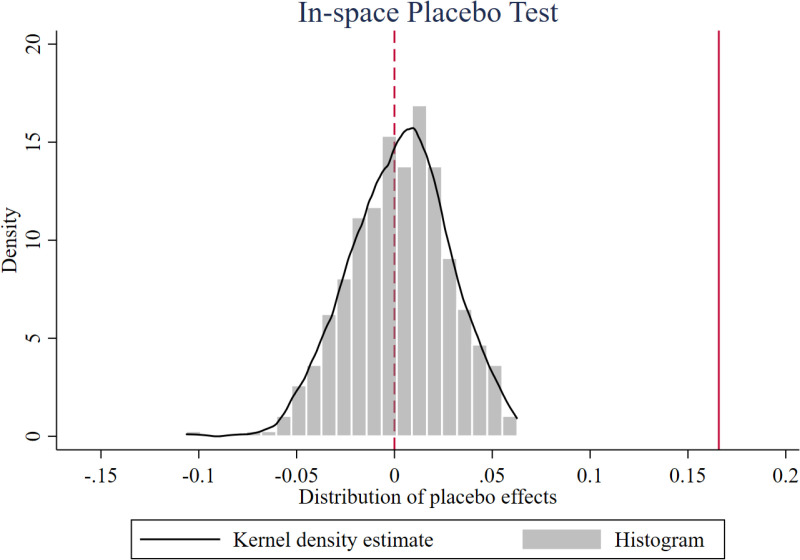
In-space placebo test. This figure shows the in-space placebo test. The two-sided p-value is 0.000***.

Second, we conduct an in-space placebo test, in which firms are randomly selected from the sample with nonreplacement to form a false treatment group for DID estimation. Fig 4 shows the distribution of placebo effects from 500 repetitions. The coefficients of the false treatment group (the kernel density curve) follow a near-normal distribution around zero, indicating no impact on firms’ EE. In contrast, the coefficients of the true treatment group (solid line on the right, i.e., benchmark analysis coefficient: 0.166) lies significantly outside this distribution, confirming that RTL’s effect on firms’ EE is robust.

### 4.5. Additional tests

#### 4.5.1. Quantile analysis.

This study adopts quantile analysis to investigate RTL’s effect on firms at different EE levels. We categorize sample firms into five groups based on their initial EE levels: 10%, 25%, 50%, 75%, and 90%. Table 7 shows that the policy effect of RTL gradually expands as the EE level increases; however, raising firms with low initial EE levels should be the main policy objective. Therefore, the authorities should appropriately adjust RTL to enhance its positive effect on firms with low EE levels.

**Table 7:** Quantile analysis.

	EE(q10)	EE(q25)	EE(q50)	EE(q75)	EE(q90)
RB*Time	0.291***	0.379***	0.436***	0.561***	0.651***
	(0.000)	(0.000)	(0.000)	(0.000)	(0.000)
Control Variables	Yes	Yes	Yes	Yes	Yes
Fixed Effect	Yes	Yes	Yes	Yes	Yes
Observations	14,198	14,198	14,198	14,198	14,198
Pseudo R-squared	0.119	0.135	0.153	0.177	0.195

This table presents the results of quantile analysis. The fixed effect model is adopted to control the time and firm fixed effect. Robust standard errors are used. ***, **, and * indicating significance at the 1%, 5%, and 10% levels, respectively.

#### 4.5.2. Mediating effect analysis.

We construct mediating effect models, i.e., Eqs 5, 6 and 7, to test Hypothesis 2: whether RTL’s effect on firms’ EE is transmitted through R&D.


$${EE}_{i,t}\;=\;\alpha_{i,t}^{2}+\;\beta_{i,t}^{1}{RB*Time}_{i,t}\;+\;\theta_{i,t}^{1}{Controls}_{i,t}\;+\;\mu_{i}{{\;+\;v}_{t}\;+\;\varepsilon }_{i,t}$$ (5)

$${R\&D}_{i,t}\;=\;\alpha_{i,t}^{2}\;+\;\beta_{i,t}^{2}{RB*Time}_{i,t}\;+\;\theta_{i,t}^{2}{Controls}_{i,t}\;+\;\mu_{i}{{\;+\;v}_{t}\;+\;\varepsilon }_{i,t}$$ (6)

$${EE}_{i,t}\;=\;\alpha_{i,t}^{3}\;+\;\beta_{i,t}^{3}{RB*Time}_{i,t}\;+\;{\delta_{i,t}R\&D}_{i,t}\;+\;\theta_{i,t}^{3}{Controls}_{i,t}\;+\;\mu_{i}{{\;+\;v}_{t}\;+\;\varepsilon }_{i,t}$$ (7)

where *R&D* is the natural logarithm of R&D investments plus one; other variables are introduced in Eq 2.

The regression results for Eqs 5, 6, and 7 are shown in Table 8. All the coefficients of RB*Time and *R&D* are significantly positive, indicating that RTL enhances resource-based firms’ EE through R&D investments. The results support Hypothesis 2 and confirm the existence of the Porter hypothesis in China.

**Table 8:** Mediating effect analysis.

	EE	R&D	EE
RB*Time	0.166***	0.358**	0.162***
	(0.000)	(0.047)	(0.000)
R&D			0.011***
			(0.000)
Control Variables	Yes	Yes	Yes
Sobel test		0.004	
		(z = 1.89; p = 0.059)	
Bootstrap test		0.162	
(Direct effect)		(z = 10.20; p = 0.000)	
Bootstrap test		0.004	
(Indirect effect)		(z = 2.43; p = 0.015)	
Fixed Effect	Yes	Yes	Yes
Observations	14,198	14,198	14,198
R-squared	0.270	0.025	0.323

This table presents the results of mediating effect analysis. The fixed effect model is adopted to control the time and firm fixed effect. Robust standard errors are used. ***, **, and * indicating significance at the 1%, 5%, and 10% levels, respectively. The Bootstrap test sampling 1000 times. *R&D* is the natural logarithm of R&D investments plus one.

#### 4.5.3. Heterogeneous impacts.

When confronted with environmental policies, firms with PC will likely be affected in two opposing ways. First, such firms possibly receive financial support from the government and thus benefit from environmental policies; second, such firms may be used as a benchmark by the authorities to enforce environmental policies and thus suffer stronger shocks. Thus, we reuses Eq 2 to examine the heterogeneous impact of the RTL on firms’ EE with PC and non-PC.

Following Wu et al. (2012), Francis et al. (2009), and Chen et al. (2011) [41–43], a PC firm is defined as its chairperson or chief executive officer serving or having served as a government official; others are non-PC firms. Table 9 shows confirming that PC firms have a greater positive impact on EE. The result demonstrates a predominance of the first effect, i.e., PC firms possibly receive financial support from the government and thus benefit from the enforcement of RTL, indicating the necessity for firms to establish PC with the government.

**Table 9:** Heterogeneous impacts: PC and non-PC.

	PC			non-PC		
	EE	EE	EE	EE	EE	EE
RB*Time	0.216***	0.218***	0.219***	0.129***	0.128***	0.145***
	(0.000)	(0.000)	(0.000)	(0.000)	(0.000)	(0.000)
Governance		Yes	Yes		Yes	Yes
Fundamentals			Yes			Yes
Fixed Effect	Yes	Yes	Yes	Yes	Yes	Yes
Observations	3,673	3,673	3,673	10,525	10,525	10,525
R-squared	0.054	0.058	0.071	0.040	0.046	0.095

This table reports the results of heterogeneous impact - PC vs. non-PC. The fixed effect model is adopted to control the time and firm fixed effect. Robust standard errors are used. ***, **, and * indicating significance at the 1%, 5%, and 10% levels, respectively. We add our proposed factors into the regression model step by step. A PC firm is defined as its chairperson or chief executive officer serving or having served as a government official; others are non-PC firms.

Besides, China is a vast country, and its resource factor endowment differs in various areas. Thus, we divide our sample into eastern, middle, and western areas and reuse Eq 2 for regression analysis. Table 10 shows that RTL has no significant impact in the western region, while its effect is significantly stronger in the middle region compared to the eastern region. This may be due to the higher proportion of resource-based firms in the middle region, along with a certain foundation of capital and technology. As a result, the RTL is more likely to stimulate innovation in the middle region, significantly improving local firm’ EE. The empirical results suggest that the central government should grant local governments greater tax administration powers, appropriately increase support for the western area, adopt differentiated policies and coordination among local governments, and enhance firms’ EE nationwide.

**Table 10:** Heterogeneous impacts: Three areas.

	Eastern			Middle			Western		
	EE	EE	EE	EE	EE	EE	EE	EE	EE
RB*Time	0.166***	0.161***	0.160***	0.205***	0.206***	0.231***	0.075	0.078	0.088
	(0.000)	(0.000)	(0.000)	(0.000)	(0.000)	(0.000)	(0.214)	(0.199)	(0.142)
Governance		Yes	Yes		Yes	Yes		Yes	Yes
Fundamentals			Yes			Yes			Yes
Fixed Effect	Yes	Yes	Yes	Yes	Yes	Yes	Yes	Yes	Yes
Observations	10,090	10,090	10,090	2,360	2,360	2,360	1,748	1,748	1,748
R-squared	0.035	0.042	0.085	0.064	0.070	0.116	0.046	0.057	0.076

This table reports the results of subsample analysis - three areas. The fixed effect model is adopted to control the time and firm fixed effect. Robust standard errors are used. ***, **, and * indicating significance at the 1%, 5%, and 10% levels, respectively. We add our proposed factors into the regression model step by step. Eastern region includes Beijing, Tianjin, Hebei, Liaoning, Shandong, Jiangsu, Zhejiang, Shanghai, Fujian, Guangdong, Guangxi and Hainan; Middle region includes Inner Mongolia, Heilongjiang, Jilin, Shanxi, Henan, Anhui, Jiangxi, Hubei and Hunan; Western region includes Xinjiang, Qinghai, Ningxia, Gansu, Shaanxi, Tibet, Sichuan, Chongqing, Guizhou and Yunnan.

## 5. Conclusion

As the largest energy producer and consumer worldwide, promoting energy utilization efficiency and sustainable development is significant to long-term social development in China. RTL is China’s first tax law on resource development, and its enforcement plays a crucial role in rational resource exploitation and utilization. To assess whether such a policy has achieved its desired goals, we utilize a DID approach and panel data of A-shares from 2017 to 2022 to explore the effect of RTL on resource-based firms’ EE.

Our benchmark analysis and robustness tests demonstrate a positive effect of RTL on resource-based firms’ EE. The parallel trend test shows that the positive policy effect emerged in 2020 and gradually increased. Furthermore, we utilize several additional tests to explore the policy effect under various situations. a) RTL boosts more for firms with higher initial EE levels than those with lower levels. b) RTL enhances EE by promoting firms’ R&D investments. Therefore, this study confirms the existence of the Porter hypothesis in China. c) The heterogeneous impacts show a stronger positive effect of RTL in the PC firms and in the middle region.

This study examines the impact of RTL on EE in resource-based firms. However, the RTL policy may lead to the exit of small and high-risk firms due to policy pressure, potentially influencing the policy effects. We do not analyze these exits separately due to the length of this study. Future research could address these areas, providing valuable insights to achieve more comprehensive conclusions.

## Supporting information

S1 Data Dataset. (ZIP)

S1 File Variable definitions. (ZIP)
