# Supplementary material for: Evidence on the Porter hypothesis: China’s Resource Tax Law may be a path to achieve corporate sustainable development
Source: PLoS One. 2025 May 30;20(5):e0323668. doi: 10.1371/journal.pone.0323668 (PMC12124751; doi:10.1371/journal.pone.0323668)
Supplement: S1 File — (ZIP) [file pone.0323668.s002.zip › S1 File/S1 File.pdf]

Supporting Information

Appendix A. Variable Definitions

Variable	Definition
EE	This is the natural logarithm of the output ratio to energy consumption plus one. Output is measured by total operating income; energy consumption stands for a firm's total consumption of standard coal, which is the sum of water consumption, electricity consumption, raw coal use, natural gas use, gasoline use, diesel use, and centralized heating multiplied by the corresponding conversion coefficients.
RB2018	This dummy variable takes a value of one for resource-based firms in 2018 and zero otherwise.
RB*Time	This dummy variable takes a value of one for resource-based firms after 2019 and zero otherwise.
RB*Time1	This dummy variable takes a value of one for resource-based firms after 2018 and zero otherwise (for the sample period of 2017–2019).
State Owner	The shareholding ratio of state-owned
FST	The ratio of the shareholding ratio of the largest shareholder to the shareholding ratio of the second to tenth largest shareholders
Duality	This dummy variable equals one if the board chair and CEO are

	the same person and zero otherwise.
Leverage	The ratio of total liabilities to total assets
Current	The ratio of current assets to current liabilities
Cash Flow	The ratio of cash and cash equivalents to total assets
R&D	The natural logarithm of R&D investments plus one
PC	This dummy variable equals one if the chair of board or CEO serves (or has served) as a government official and zero otherwise.
